# Methylmercury formation in biofilms of *Geobacter sulfurreducens*

**DOI:** 10.3389/fmicb.2023.1079000

**Published:** 2023-01-13

**Authors:** Elena Yunda, Mareike Gutensohn, Madeleine Ramstedt, Erik Björn

**Affiliations:** Department of Chemistry, Umeå University, Umeå, Sweden

**Keywords:** methylmercury, biofilms, *Geobacter sulfurreducens*, mercury methylation, methylation rate

## Abstract

**Introduction:**

Mercury (Hg) is a major environmental pollutant that accumulates in biota predominantly in the form of methylmercury (MeHg). Surface-associated microbial communities (biofilms) represent an important source of MeHg in natural aquatic systems. In this work, we report MeHg formation in biofilms of the iron-reducing bacterium *Geobacter sulfurreducens*.

**Methods:**

Biofilms were prepared in media with varied nutrient load for 3, 5, or 7 days, and their structural properties were characterized using confocal laser scanning microscopy, cryo-scanning electron microscopy and Fourier-transform infrared spectroscopy.

**Results:**

Biofilms cultivated for 3 days with vitamins in the medium had the highest surface coverage, and they also contained abundant extracellular matrix. Using 3 and 7-days-old biofilms, we demonstrate that *G. sulfurreducens* biofilms prepared in media with various nutrient load produce MeHg, of which a significant portion is released to the surrounding medium. The Hg methylation rate constant determined in 6-h assays in a low-nutrient assay medium with 3-days-old biofilms was 3.9 ± 2.0 ∙ 10^−14^  L ∙ cell^−1^ ∙ h^−1^, which is three to five times lower than the rates found in assays with planktonic cultures of *G. sulfurreducens* in this and previous studies. The fraction of MeHg of total Hg within the biofilms was, however, remarkably high (close to 50%), and medium/biofilm partitioning of inorganic Hg (Hg(II)) indicated low accumulation of Hg(II) in biofilms.

**Discussion:**

These findings suggest a high Hg(II) methylation capacity of *G. sulfurreducens* biofilms and that Hg(II) transfer to the biofilm is the rate-limiting step for MeHg formation in this systems.

## 1. Introduction

Methylmercury (MeHg) is a neurotoxic compound that is formed as a result of methylation of divalent inorganic mercury (Hg(II)) by microorganisms carrying the genes *hgcA* and *hgcB* (Parks et al., 2013; Regnell and Watras, 2019). The formation of MeHg occurs in oxygen-depleted zones of wetlands (Tjerngren et al., 2012; Liem-Nguyen et al., 2021), sediments (Jonsson et al., 2014; de Oliveira et al., 2015; Bigham et al., 2017; Jones et al., 2020), rice paddy soils (Qin et al., 2020; Wang et al., 2021) and fresh-and marine water columns (Capo et al., 2020). The bioaccumulation, trophic transfer efficiency and very low elimination rate lead to a biomagnification of MeHg in aquatic food webs and potentially exposure of wildlife and humans to elevated MeHg concentrations (Tollefson and Cordle, 1986; Orihel et al., 2007). Metagenome research (Podar et al., 2015; Capo et al., 2020; Peterson et al., 2020), sequencing studies (Bravo et al., 2018; Xu et al., 2021) and experimental incubations (Gilmour et al., 2013; Bravo et al., 2018) have revealed diverse bacterial communities to be responsible for Hg methylation including sulfate-reducing bacteria, iron-reducing bacteria, and methanogens. Several bacterial isolates were used as model organisms in studies of mechanistic principles of Hg methylation covering the aspects of Hg speciation in the medium (Schaefer et al., 2011; Thomas et al., 2014, 2018; Isaure et al., 2020), its coordination environment on bacterial cell membranes (Song et al., 2020; Thomas et al., 2020) and the microbial uptake of Hg (Schaefer et al., 2011; Thomas et al., 2014; Szczuka et al., 2015; Wang et al., 2020), among others. These works allowed for significant advancements in understanding which factors control the rate and amount of MeHg formation. Yet the still existing inaccuracy of predictive models for MeHg formation in the environment suggests that further developments in the experimental designs are necessary.

One experimental challenge in mechanistic studies of Hg methylation is to control the speciation of Hg and the metabolic state of microorganisms. Both these factors can contribute to changes in the rate of MeHg formation (Goñi-Urriza et al., 2015). Many studies have adopted a two-step experimental protocol, in which the growth of bacterial cells in a suitable nutritive medium is followed by washing and resuspending a certain number of cells in a nutrient-poor and chemically defined assay buffer for an incubation period of several hours (Schaefer et al., 2011; Thomas et al., 2014, 2018, 2020; Szczuka et al., 2015; Wang et al., 2021). These conditions allow chemical speciation and cell MeHg concentration to be monitored while the growth of cells and metabolic changes are fairly limited. Furthermore, the homogeneity in properties and functions of bacterial cells in a culture is an important assumption for normalization of values of MeHg formation rate per cell. Hence, laboratory experiments were conducted almost exclusively (Lin and Jay, 2007; Lin et al., 2013) on planktonic cell cultures. In contrast, in natural environments microorganisms are predominantly present in aggregated communities called biofilms (Flemming and Wuertz, 2019). In sediments and aquifers, for example, the number of cells in biofilms is estimated to be 100–1,000 times higher than that of unattached cells (Flemming and Wuertz, 2019).

Biofilms are distinct from planktonic cells due to the presence of an accumulated extracellular matrix that links cells and shapes the structure of the biofilm (Flemming et al., 2016). The matrix is composed of polymeric substances enabling accumulation of various substances from the bulk medium surrounding the biofilm, as well as substances produced by bacterial cells or that are a result of cell degradation within the biofilm volume. Several field studies reported MeHg and Hg(II) accumulation in freshwater biofilms grown in rivers (Dominique et al., 2007; Dranguet et al., 2017; Leclerc et al., 2021), creeks (Olsen et al., 2016; Schwartz et al., 2019), and lakes (Leclerc et al., 2015; Bouchet et al., 2018), and biofilms represent a MeHg source for macroinvertebrates and fish (Dominique et al., 2007; Cremona et al., 2009). Biofilms can accumulate low-molecular-weight thiol compounds (Leclerc et al., 2015; Bouchet et al., 2018), some of which are known to promote microbial Hg uptake and methylation (Schaefer and Morel, 2009). However, there are noteworthy few mechanistic studies of Hg methylation in controlled laboratory experiments on biofilms from bacterial isolates. One laboratory study reported 10 times higher Hg methylation rates in biofilms compared to planktonic cultures of a sulfate-reducing bacterium (Lin and Jay, 2007), and this result was suggested to be due to metabolic differences between the two cultures (Lin et al., 2013). It is clear that understanding the processes controlling Hg availability and methylation in biofilms is important and will require controlled studies on biofilms of model organisms.

Iron-reducing bacteria are important members of Hg-methylating communities in natural environments (Bravo et al., 2018; Leclerc et al., 2021) and one common bacterium used to study mechanisms of MeHg formation in laboratory settings is *Geobacter sulfurreducens* (Kerin et al., 2006; Schaefer and Morel, 2009; Schaefer et al., 2014; Lin et al., 2015; Si et al., 2015; Qian et al., 2016; Thomas et al., 2020). Geobacter species generally play an important role in the biogeochemistry of many environments due to their capacity to grow on abundant minerals containing iron or manganese, and interact with other trace metals such as uranium or vanadium (Cologgi et al., 2014; Yan et al., 2022). The use of *G. sulfurreducens* enables relatively good control of Hg speciation and its availability for cellular uptake, because it is incapable of sulfate reduction, thus, minimizing the formation of sulfides during the assay (Schaefer and Morel, 2009). Notably, *G. sulfurreducens* forms biofilms in a variety of settings including iron minerals (Reguera et al., 2007; Wilkins et al., 2007; Downie et al., 2018; Newsome et al., 2018), poised electrodes (Steidl et al., 2016; Li et al., 2017; Chadwick et al., 2019), and glass (Reguera et al., 2007; Klimes et al., 2010; Cologgi et al., 2014; Richter et al., 2017), making it a promising model organism for studying Hg methylation in bacterial biofilms.

The overall purpose of this work was to establish optimal conditions for Hg methylation assays with *G. sulfurreducens* biofilms and to quantify Hg methylation and partitioning in these systems. Structural changes in *G. sulfurreducens* biofilms were monitored as a function of growth conditions and Hg(II) methylation rate and partitioning of MeHg and Hg(II) were determined in selected biofilm assays. Biofilms were prepared in media with varied nutrient load by addition of yeast extract or a mixture of vitamins for 3, 5, or 7 days, followed by exposure to Hg(II). The yeast extract is known to promote cell growth in *G. sulfurreducens* planktonic cultures (Coppi et al., 2001), and it was added to investigate whether this rich medium also promotes the growth of cells in a biofilm form. Vitamins are commonly used in preparation of nutritive media in studies of *G. sulfurreducens* biofilms (Reguera et al., 2007; Cologgi et al., 2014), but are usually not present in standard nutritive media in Hg methylation studies. Using these two amendments we thus aimed to investigate what conditions allow abundant biofilm growth together with the capacity to methylate Hg. The biochemical composition and structural properties of the biofilms were examined using infrared spectroscopy, confocal laser scanning microscopy and cryo-scanning electron microscopy. By carefully analyzing several structural characteristics of biofilms, as well as methylation of Hg(II) and partitioning of Hg(II) and MeHg in biofilms, we contribute to the advancement of the mechanistic understanding of Hg(II) transformations in environmental systems and pave the way for future studies of Hg methylation in *G. sulfurreducens* biofilms.

## 2. Materials and methods

### 2.1. Bacterial strain and culture conditions

*Geobacter sulfurreducens* PCA (ATCC 51573) was purchased from DSMZ (Germany) and used in all experiments. The bacterial growth was routinely maintained as a liquid culture under N_2_ atmosphere at 30°C and pH 6.8. The growth medium (hereafter called standard nutritive medium) contained 10 mM sodium acetate, 40 mM sodium fumarate, 10 mM MOPS, 5.6 mM NH_4_Cl, 1.3 mM KCl, 0.2 mM NaCl, 0.1 mM MgSO_4_, 8.8 μM CaCl_2_, 0.05 mM NaH_2_PO_4_, 1% (v/v) Wolfe’s trace metals solution containing 10 times lower concentration of CuSO_4_, 0.6 μM Na_2_SeO_3_, and 1 μg/mL resazurin (Schaefer and Morel, 2009). Resazurin was used as an indicator of the redox status of the medium containing bacterial cells. The growth of bacterial cultures was monitored using optical density measurements of cultures at 660 nm (UV-1201 spectrophotometer, Shimadzu). The late-exponential-phase cultures were obtained within 40–44 h after bacterial inoculation (1%–2%) into the fresh standard nutritive medium (Supplementary Figure S1a).

### 2.2. Set-up for biofilm formation

The late-exponential-phase cultures were diluted in three different nutritive media containing vertically placed acid-washed (15% HCl) glass substrates with the surface area of ~4 × 1 cm^2^, prepared by cutting microscopy slides (ABAA000001##02E, Thermo Scientific). The starting optical density of bacterial cultures used for the biofilm growth was ~0.02 and the volume of the nutritive media was 15 mL. The nutritive media used were (i) standard nutritive medium, (ii) standard nutritive medium amended with yeast extract (1 g/L, Merck), or (iii) standard nutritive medium amended with vitamins mixture (1% v/v, MD-*VS*™, ATCC). The standard nutritive medium composition was selected based on previous studies and has been optimized for *G. sulfurreducens* Hg methylation assays (Schaefer and Morel, 2009; Schaefer et al., 2011). Biofilms were cultivated for 3, 5, or 7 days, in static conditions and in dark at 30°C. When biofilms were cultivated for 5 or 7 days, every other day ~7 mL of the nutritive medium was carefully replaced with the fresh corresponding medium using N_2_-filled syringe to support continuing bacterial growth, as schematically presented in Supplementary Figure S1b. The range of conditions for biofilm formation experiments was selected based on earlier research of *G. sulfurreducens* biofilms grown on glass (Reguera et al., 2007; Cologgi et al., 2014), as well as a study of Hg methylation by biofilms in laboratory settings (Lin and Jay, 2007).

### 2.3. Biofilm morphology

Morphological properties of biofilms were studied using confocal laser scanning microscopy (CLSM). Biofilms were carefully rinsed twice by dipping glass slides into cell-free nutritive media. The nutritive medium (100 μL) containing 1.5 μL of SYTO 9 stock solution, prepared by 10-fold dilution of SYTO 9 dye (3.34 mM in DMSO, Invitrogen) in 0.9% (w/v) NaCl, was spread on top of the glass slide with the biofilm. Biofilms were stained in dark for 15 min, followed by removal of dye excess by dipping slides in cell-free nutritive media. Glycerol solution (50% in milli-Q water) was used to mount glass cover slips on top of the biofilms for subsequent observation with the microscope. Images were recorded every 1.74 μm along the biofilm height using a DIC 20X (NA 0.75) objective of a Nikon A1R confocal microscope controlled by Nikon NIS Elements interface. The percent of the surface covered with bacteria was determined using a function of manual threshold adjustment in Fiji (Schindelin et al., 2012) on two-dimensional images representing maximal intensity projections. The structural parameters of biofilms were calculated using BiofilmQ software following segmentation of three-dimensional biofilm images in cubes (pseudo-cells) with the side length of 1.87 μm after semi-manual threshold adjustment (Hartmann et al., 2021). The resulted number of pseudo-cells per image field was used for estimation of the approximate number of bacterial cells in biofilms (Hartmann et al., 2021) in Hg methylation assays after adjustment of the value to the surface area of the glass slides. The estimation of cell number was made with the assumption that the two sides of the glass slides were equally covered with the biomass since the slides were placed vertically during the incubation. In addition, cell numbers were estimated assuming that biomass distribution recorded in the images is representative of full glass slide area. Four to seven images collected on samples from two separate experiments were used for the analysis of structural parameters of biofilms. Biofilms biomass was estimated as a ratio of biofilms biovolume (sum of all segmented cubes) to the substrate surface area. The roughness of biofilms was determined as the mean of the difference between local thickness (one cube area) and mean thickness of the biofilm divided by mean thickness of the biofilm (Hartmann et al., 2021). Statistically significant changes were determined using the Student’s *t*-test in Microsoft Excel.

### 2.4. Examination of biofilm extracellular matrix

Biofilms were prepared as described above, except that ~1 × 1 cm^2^ glass slides were placed in 6 mL of the nutritive medium for cultivation. Prior the analysis, biofilms were rinsed by dipping the glass slides twice into the assay buffer (the assay buffer was the same as for Hg methylation assay, but not amended with Hg), plunged into liquid nitrogen slush, sublimated *in vacuo* for 30 min at-90°C, and coated with a thin layer of platinum. Imaging was performed on a Carl Zeiss Merlin field-emission cryogenic scanning electron microscope (cryo-FESEM), fitted with a Quorum Technologies PP3000T cryo preparation system. Images were taken at-140°C using secondary electron detectors at an accelerating voltage of 3 kV and a probe current of 50 pA. The images presented are representative from a series recorded on duplicate samples.

### 2.5. Biochemical composition of biofilms

Attenuated total reflectance-Fourier transform infrared (ATR-FTIR) spectroscopy was used to characterize molecular composition of biofilms. Biofilms were scraped off the glass slides using pipette tips and placed on the diamond crystal in the ATR accessory of the Bruker Vertex 80v FTIR spectrometer. Each spectrum was recorded by collecting 100 scans between 4,000 and 700 cm^−1^. The resolution of the single beam was 4 cm^−1^. The absorbance scale of the spectra correspond to log (R_reference_/R_sample_), where R is the internal reflectance. A spectrum of cell-free medium was used as a reference for the biofilm spectra. Spectra were processed to remove water vapor contribution and baseline-corrected at 3,580, 2,750, 1,800, and 900 cm^−1^ in OPUS 7.8 software.

### 2.6. Hg methylation assay

In a glovebox maintained under N_2_ atmosphere (Saffron Scientific Equipment Ltd., North Yorkshire, United Kingdom), biofilms were carefully rinsed twice as described above and introduced into 6 mL of the assay buffer containing 100 nM Hg(II), prepared by dilution of 5 mM Hg(NO_3_)_2_ stock solution (28941-100ML-F, Fluka). The assay buffer contained 1 mM acetate, 1 mM fumarate, 10 mM MOPS, 0.1 mM NH_4_Cl, 1.3 mM KCl, 0.15 mM MgSO_4_, 5 mM NaH_2_PO_4_, 0.17 mM NaCl, and 1 mg/L resazurin (Schaefer et al., 2014). The assay buffer was pre-equilibrated with Hg(II) for ~1 h before incubation with biofilms. The assays were carried out at 30°C in dark in acid-cleaned glass vials covered with Teflon stoppers and sealed with crimps. After 6 h of incubation, samples were collected and spiked with Me^200^Hg or ^200^Hg for quantification by isotope dilution analysis of MeHg and total Hg, respectively. Inorganic Hg was determined by subtraction of MeHg from total Hg. Analyses were performed on biofilm and biofilm-surrounding media samples. Samples of the medium surrounding biofilms were prepared by taking 1 mL aliquot of the solution surrounding a glass slide with the biofilm, after which the glass slide was carefully extracted from the vial for collecting the biofilm sample. Biofilms were scraped off the glass slides into 1 mL of the assay buffer using inoculating loops and vortexed before splitting the samples in two for MeHg and total Hg analyses. Biofilms and media collected for MeHg analyses were processed for 24 h under alkaline conditions by addition (1:10 v/v) of 25% (w/v) NaOH, whereas samples collected for total Hg quantification were treated with BrCl following the EPA 1631E method (Method 1631, Revision E: Mercury in Water by Oxidation, Purge and Trap, and Cold Vapor Atomic Fluorescence Spectrometry. EPA-821-R-02-019, 2002). Samples were stored at −20°C until analysis. If not stated otherwise, three replicate samples were prepared and statistically significant difference between conditions was determined using the Student’s *t*-test in Microsoft Excel.

Separately from biofilm experiments, Hg methylation assays were performed with *G. sulfurreducens* planktonic cultures in vials of the same volume and with the same assay medium as for biofilm experiments. Planktonic cultures used for the assay were grown until late-exponential phase in the standard nutritive medium amended with the vitamins mixture (1% v/v), washed twice with the assay buffer and transferred to the assay buffer amended with 100 nM Hg(II) at the OD of ~0.01. The conditions of the assay and the quantification of MeHg and total Hg were identical to biofilm assays. Hg(II) associated with cells was determined by subtraction of MeHg from total Hg values that were obtained by calculating the difference of corresponding values for samples of bacterial suspensions and samples that were filtered prior to the analysis.

### 2.7. Total Hg and MeHg analyses

MeHg concentration in biofilms and biofilm-surrounding media was determined using thermal desorption gas chromatography−ICPMS (TD-100 Markes – GC 7890B Agilent – ICP-MS 7700 Agilent) after adjustment of samples pH to ~4.5 using 5 M HCl and 2 M CH_3_COONH_4_. Samples were derivatized with NaB(C_2_H_5_)_4_, purged with N_2_ and trapped on Tenax adsorbent. Total Hg concentration was determined using CETAC HGX-200 cold vapor system coupled with ICPMS (8900 Agilent) after sample neutralization with NH_2_OH∙HCl and online Hg(II) reduction with SnCl_2_ (Adediran et al., 2019).

## 3. Results and discussion

**Figure 1:**
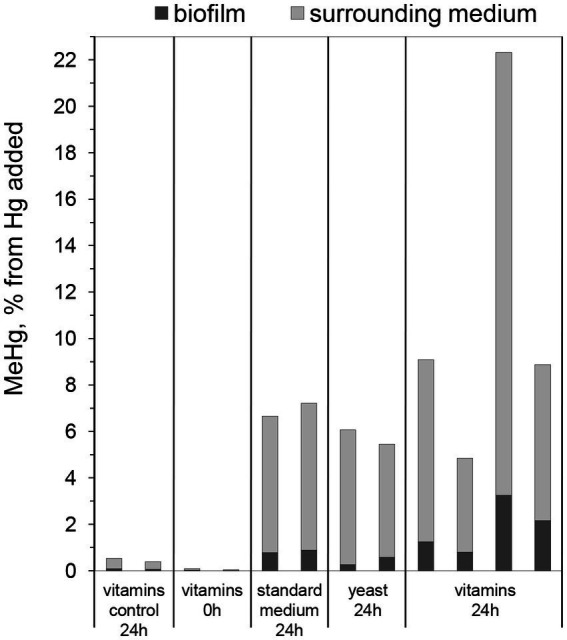
Percentage of MeHg produced from 100 nM added Hg, determined in biofilms and surrounding media, after 0 or 24 h of incubation with *Geobacter sulfurreducens* biofilms grown for 7 days in standard nutritive medium (“standard medium”), in standard nutritive medium amended with yeast extract (“yeast”), or standard nutritive medium amended with vitamins mixture (“vitamins”). Control represents samples without Hg addition. Bars represent MeHg produced of which the gray part represent MeHg in the surrounding medium and the black part MeHg in the biofilm. Each bar represents one experimental replica.

In a first pilot study, Hg methylation assays were performed using 7-day-old biofilms cultivated in three different media, i.e., (i) standard nutritive medium, (ii) standard nutritive medium amended with yeast extract, and (iii) standard nutritive medium amended with vitamins mixture (as described further in the Materials and methods section). It was found that all biofilms produced MeHg with on average ~ 8% of the added Hg(II) transformed into MeHg over a time period of 24 h (Figure 1). This result shows that all studied nutritive media support the growth of biofilms with cells capable of Hg methylation. Furthermore, the fraction of MeHg associated with biofilms was from 7 to 20% of the total produced MeHg for the three different cultivated biofilms. The substantial portion of MeHg in the medium surrounding biofilms supports the notion that *G. sulfurreducens* exports MeHg from the intracellular cell compartments during methylation assays (Schaefer et al., 2011).

**Figure 2:**
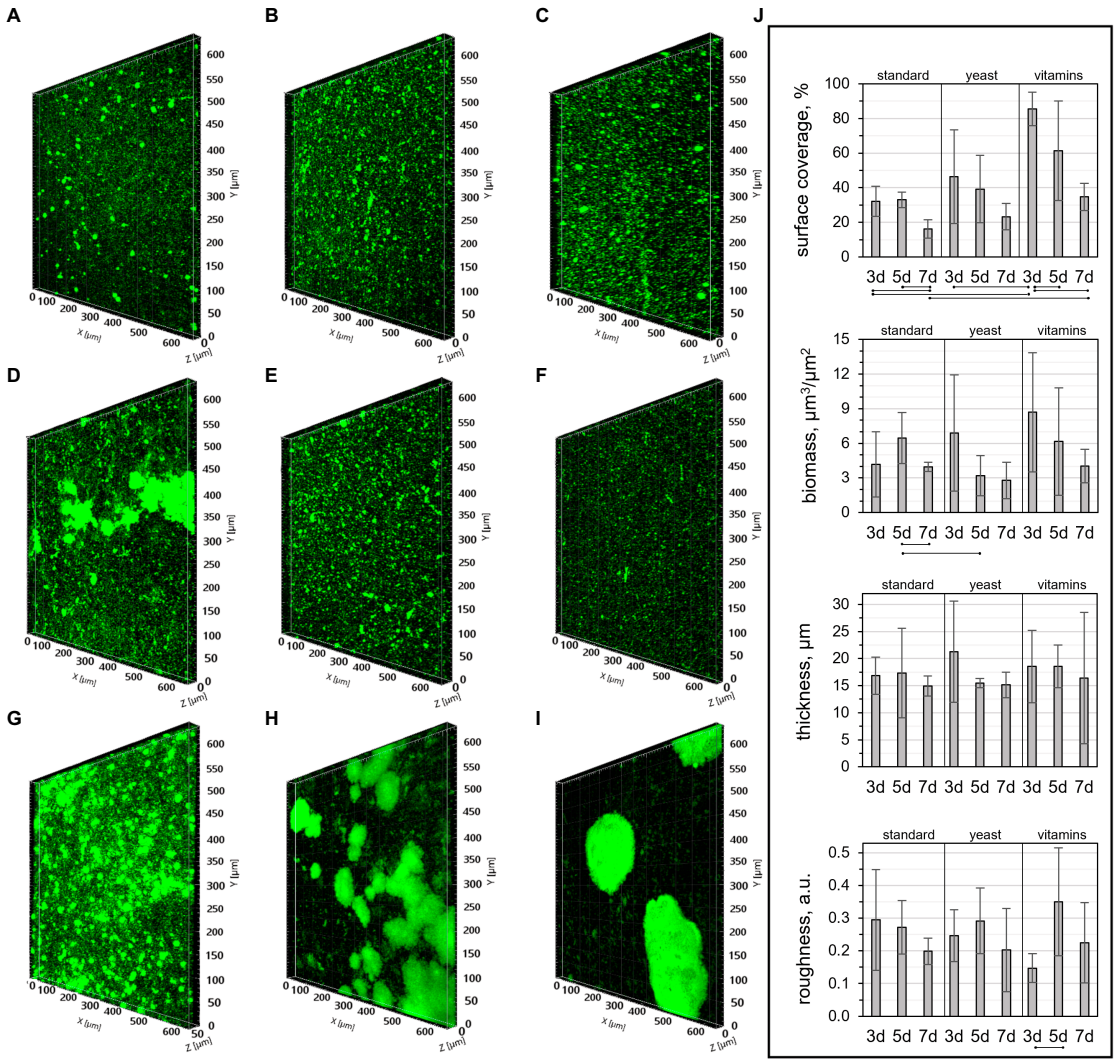
CLSM images of biofilms cultivated in **(A-C)** standard nutritive medium, **(D-F)** standard nutritive medium amended with yeast extract, and **(G-I)** standard nutritive medium amended with vitamins mixture for **(A,D,G)** three, **(B,E,H)** five or **(C,F,I)** 7 days with **(j)** corresponding structural parameters. The lines indicate statistically significant differences (*p* ≤ 0.05) for samples prepared in the same medium, or cultivated for the same number of days.

To get a better understanding of biofilm growth patterns and the evolution of structural properties as a function of time, CLSM images of biofilms cultivated for 3, 5, or 7 days in the three nutritive media were taken (Figure 2). After 3 days of growth in standard nutritive medium, cells could be seen dispersed in a ~17 μm thick layer covering ~32% of the substrate surface (Figures 2A,J). Within this layer, sphere-shaped cell colonies were distributed. Colony formation in biofilms is one strategy utilized by bacteria to optimize access to nutrients (Stoodley et al., 2002), while its multilayer structure is likely associated with the presence of extracellular matrix binding the cells (Karatan and Watnick, 2009). In 5- and 7-day-old biofilms, the appearance of colonies was less distinct (Figures 2B,C). The variation in colony morphology did not promote a significant change in biofilm roughness or thickness, but the surface coverage and biomass were lower in 7-day-old biofilms (Figure 2J). The size of colonies was on average larger in biofilms grown with yeast extract for 3 days (Figure 2D), but the biomass decreased after 5 days of growth, which was reflected by a decrease of colony size in older biofilms (Figures 2E,F). It should be noted that three-dimensional structures loosely associated with glass surfaces were observed by naked eye when biofilms were grown in medium with yeast extract for 7 days. These structures were weakly bound to surfaces and, thus, were easily washed off during sample handling following biofilm growth. The large bacterial clusters associated with glass surfaces were also observed in medium with vitamins. They were clearly visible in CLSM images (Figures 2H,I), which indicates a higher strength of attachment when grown in presence of vitamins. Indeed, these enhanced interactions with the substrate surface may explain the higher biofilm content in the 3-day-old biofilm grown in medium with vitamins, where ~85% of the surface was covered by bacteria (Figures 2G,J). Overall, the CLSM results suggest that higher nutrient content does not promote a significant increase in biofilm biomass.

Structural parameters of 3-day-old biofilms grown with vitamins were remarkably consistent with those reported previously for a biofilm of the same age and cultivated in a similarly composed medium (Cologgi et al., 2014). The surface coverage, biomass, and mean thickness found in our work were 86 ± 10%, 8.9 ± 5.2 μm^3^/μm^2^, and 19 ± 7 μm, while those reported by Cologgi et al. (2014) were 92 ± 7%, 10.6 ± 3.3 μm^3^/μm^2^, and 14 ± 4 μm. However, the roughness of biofilms in this work was lower than in the work of Cologgi et al. (0.1 ± 0.04 and 0.24 ± 0.06, respectively).

**Figure 3:**
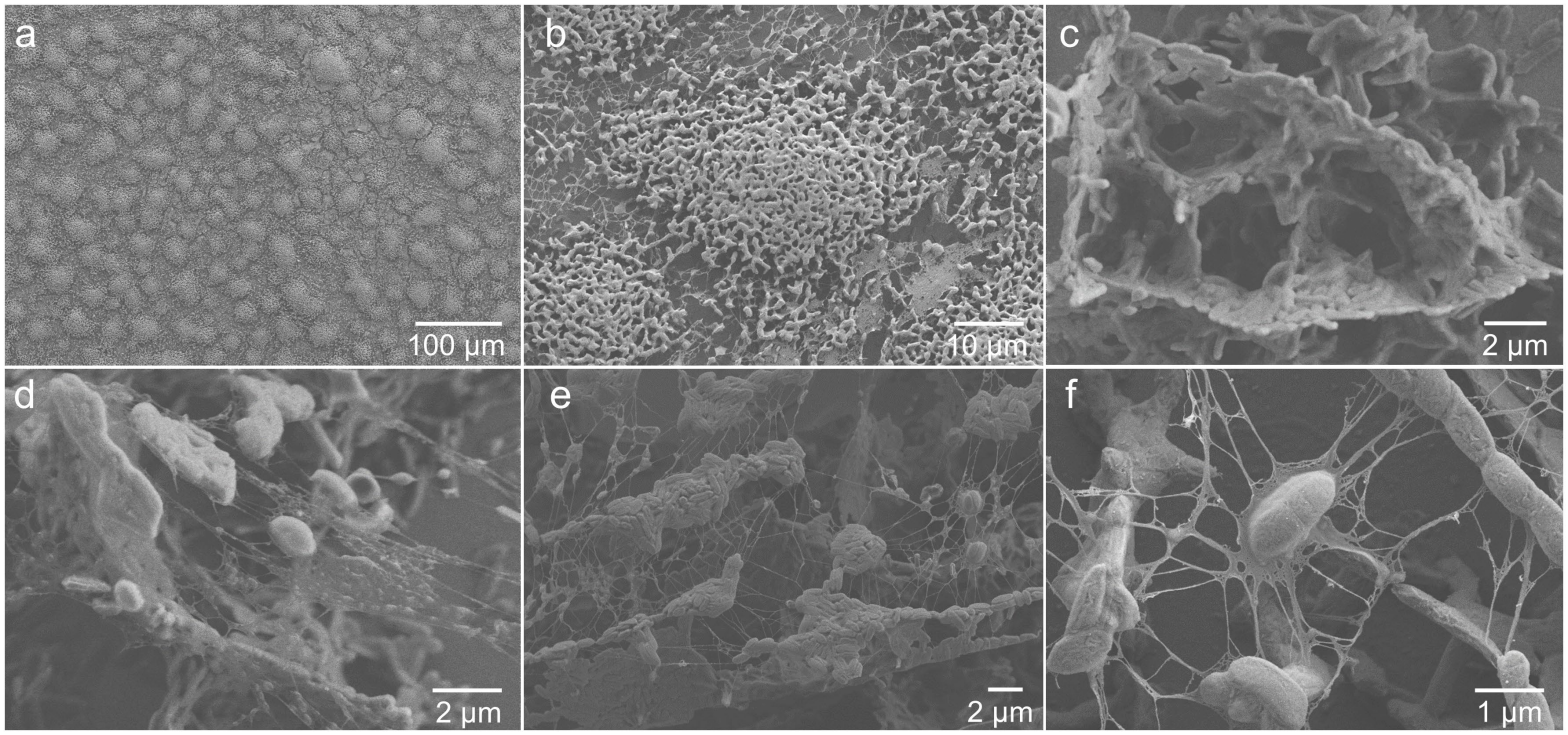
Cryo-SEM images showing extracellular matrix surrounding **(A,B)** colonies and **(D-F)** individual cells, as well as **(C)** an example of cell organization in a colony, of 3-day-old *G. sulfurreducens* biofilm grown in standard nutritive medium amended with vitamins.

To image the extracellular matrix at a higher spatial resolution, a 3-day-old biofilm grown in the medium with vitamins was observed using cryo-SEM. Figures 3A,B shows that the overall structure of the biofilm is formed by cell colonies homogeneously distributed over the surface, in agreement with the CLSM results. As shown in Figure 3C, the colonies appeared to be fairly porous, perhaps to enable storage and transport of bacterial metabolites and nutrients (Quan et al., 2022). Bacterial colonies, as well as individual cells, were clearly enveloped in extracellular material (Figures 3D–F). Furthermore, ~100–300 nm large matrix-associated bundles were detected in some places of the biofilm (Figures 3D,E). The evidence of abundant extracellular matrix is in accordance with previous studies that have demonstrated extracellular substances linking *G. sulfurreducens* cells in biofilms (Rollefson et al., 2011; Cologgi et al., 2014).

**Figure 4:**
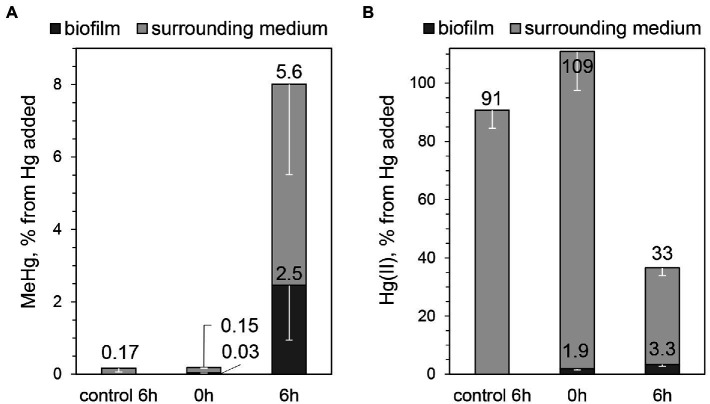
Percentage of **(A)** MeHg produced from 100 nM added Hg, determined in biofilms and surrounding media, after 0 or 6 h of incubation with *G. sulfurreducens* biofilms grown for 3 days in standard nutritive medium amended with vitamins mixture, and **(B)** Hg(II) present in biofilms and surrounding media at the same assays. Error bars indicate standard deviation obtained from three replicates.

We pursued the study of Hg methylation using 3-day-old biofilms prepared in medium with vitamin mixture since these conditions resulted in the highest surface coverage of biofilm. MeHg and Hg(II) were quantified both in biofilms and in surrounding media after 6 h of incubation with 100 nM Hg(II). Similar to the pilot assays with 7-day-old biofilms, ~8% of the initially added Hg(II) was methylated (Figure 4A). Using the average estimated cell number from CLSM, (2.0 ± 1.1) ∙ 10^9^ cells in the biofilm, the Hg methylation rate constant was calculated and found to be k_m_ = (3.9 ± 2.0) ∙ 10^−14^ L ∙ cell^−1^ ∙ h^−1^. This result is comparable to the study of biofilms of a sulfate-reducing bacterium (Lin and Jay, 2007; 3.0 ∙ 10^−13^ L ∙ cell^−1^ ∙ h^−1^, as recalculated from per-day value), although MeHg was quantified only in media surrounding the biofilms in that study. Furthermore, the k_m_ value determined for *G. sulfurreducens* biofilms in our study is ~20% of the Hg methylation rate constant obtained in *G. sulfurreducens* planktonic cultures after 6 h of incubation in the medium of the same composition as for biofilm assays, k_m_ = (2.6 ± 0.3) ∙ 10^−13^ L ∙ cell^−1^ ∙ h^−1^. Previously reported Hg methylation rate constants in planktonic culture assays with *G. sulfurreducens* vary depending on for example the type of added ligands, particularly low-molecular-weight thiols (Schaefer et al., 2011; Adediran et al., 2019). The k_m_ value found for biofilms in our study is ~20% and 40% of k_m_ values reported previously for planktonic cells for one-and two-hour-long assays without thiols addition ((2.1 ± 0.7)∙ 10^−13^ L ∙ cell^−1^ ∙ h^−1^ (Schaefer et al., 2014) and (1.0 ± 0.4) ∙ 10^−13^ L ∙ cell^−1^ ∙ h^−1^ (Schaefer et al., 2011), respectively). We therefore conclude that in the minimal nutritive conditions of the assay medium *G. sulfurreducens* biofilms methylate Hg at a lower rate compared to planktonic cultures, albeit within the same order of magnitude.

It should be noted that no cell detachment was observed during the assay (OD_660_ ≤ 0.003 of the medium surrounding biofilms after 6-h incubation). This means that all MeHg produced can be attributed to the activity of bacterial cells in the biofilms and not to planktonic cells. In biofilms, bacterial cells can vary in metabolic state depending on their location due to differences in the microenvironment of each cell. For example, lower nutrient availability and accumulation of cell metabolites can lead to a slower growth rate and death of cells in the inner and bottom parts of the biofilm (Mah and O’Toole, 2001; Karatan and Watnick, 2009). Therefore, it is possible that not all cells in the biofilm participate in Hg methylation to the same extent. Furthermore, considering Hg(II) diffusion into the biofilm, it is likely that the cells at the top of the biofilms are more exposed to Hg(II) and may be more active in methylation. Interestingly, we found low accumulation of Hg(II) in biofilms. While the partitioning coefficient “cells/medium” at 6 h of incubation for MeHg was equal to 0.41 ± 0.16, the corresponding partitioning for Hg(II) was only 0.10 ± 0.02 (Figure 4B). The low concentration of Hg(II) in biofilms could be explained by a slow mass transfer of Hg(II) into the biofilm, in comparison with planktonic cell cultures. Indeed, the partitioning coefficient “cells/medium” for Hg(II) in planktonic cultures was consistently higher compared to biofilm assays (0.61 ± 0.18 and 0.75 ± 0.41 after 2 and 6 h of incubation for planktonic cells, compared to 0.039 ± 0.001 and 0.10 ± 0.02 after 2 and 6 h for biofilms). In planktonic cultures cells are distributed more homogeneously in solution, allowing exposure to Hg(II) from every direction, which may explain higher amount of Hg(II) associated with planktonic *G. sulfurreducens* cells.

The fraction of MeHg of total Hg in the biofilm was noteworthy high, close to 50% (2.5% and 3.3% of initially added Hg was found in biofilms as MeHg and Hg(II), respectively, Figures 4A,B). For comparison, the proportion of MeHg to total Hg was reported to be <1% in biofilms grown on artificial substrata in reservoirs of a Hg-contaminated river (Dranguet et al., 2017), <0.1% in an industrially contaminated freshwater creek (Schwartz et al., 2019), 11%–18% in a river affected by run-of-river power plants and artificial wetlands (Leclerc et al., 2021), as well as 12% and 57% in plant-associated biofilms in lake Titicaca (Bouchet et al., 2018). Experiments with planktonic cell cultures of *G. sulfurreducens* have previously shown that MeHg is not accumulated inside cells (Schaefer et al., 2011). Hence, we hypothesize that in our study MeHg in biofilms was associated with bacterial cell walls and with the biofilm matrix. The high fraction of MeHg of total Hg in biofilms reflects a remarkably high capacity of Hg methylation by *G. sulfurreducens* cells in biofilm systems. Together with the low partitioning of Hg(II) to biofilm cells these results suggest that Hg methylation in biofilms is rate-limited by the mass transfer of Hg(II) into the biofilm.

It should be noted that a substantial decrease of Hg(II) in the medium was observed during the methylation assays (Figure 4B). Hg(II) loss could occur due to Hg(II) adsorption on glass vial walls and microbial Hg reduction. This would also affect the overall mass balance of total Hg and may explain why only 44% was recovered after 6 h. Previous studies have reported an abundance of redox active proteins (cytochromes) in the extracellular matrix of *G. sulfurreducens* biofilms (Rollefson et al., 2011; Steidl et al., 2016). Furthermore, a correlation has been shown between abundance of cytochromes and Hg reduction rates (Lin et al., 2014; Qian et al., 2016). It is thus possible that Hg(II) reduction and methylation were two competing processes upon Hg(II) entering into the biofilm. In previous studies with *G. sulfurreducens* planktonic cultures the extent of Hg reduction decreased by the adsorption of Hg onto bacterial cell wall surfaces, particularly *via* binding to thiol groups (Hu et al., 2013; Lin et al., 2014). Since Hg(II) did not accumulate in the biofilm significantly but its loss in the system was high, one could assume high reduction relative to cell adsorption of Hg. Thiol groups are present on the bacterial cell wall, but they could also be accumulated in the extracellular matrix of the biofilm. This could particularly be expected for *G. sulfurreducens* biofilms, since its planktonic counterparts can produce up to ~50 nM of extracellular low molecular mass thiols compounds within 6 h at 1.1 × 10^8^ cells mL^−1^ under the same nutritive conditions of the assay medium as used in this study (Adediran et al., 2019). It will thus be highly interesting to investigate both Hg reduction and possible thiol production and accumulation in *G. sulfurreducens* biofilms in future studies.

**Figure 5:**
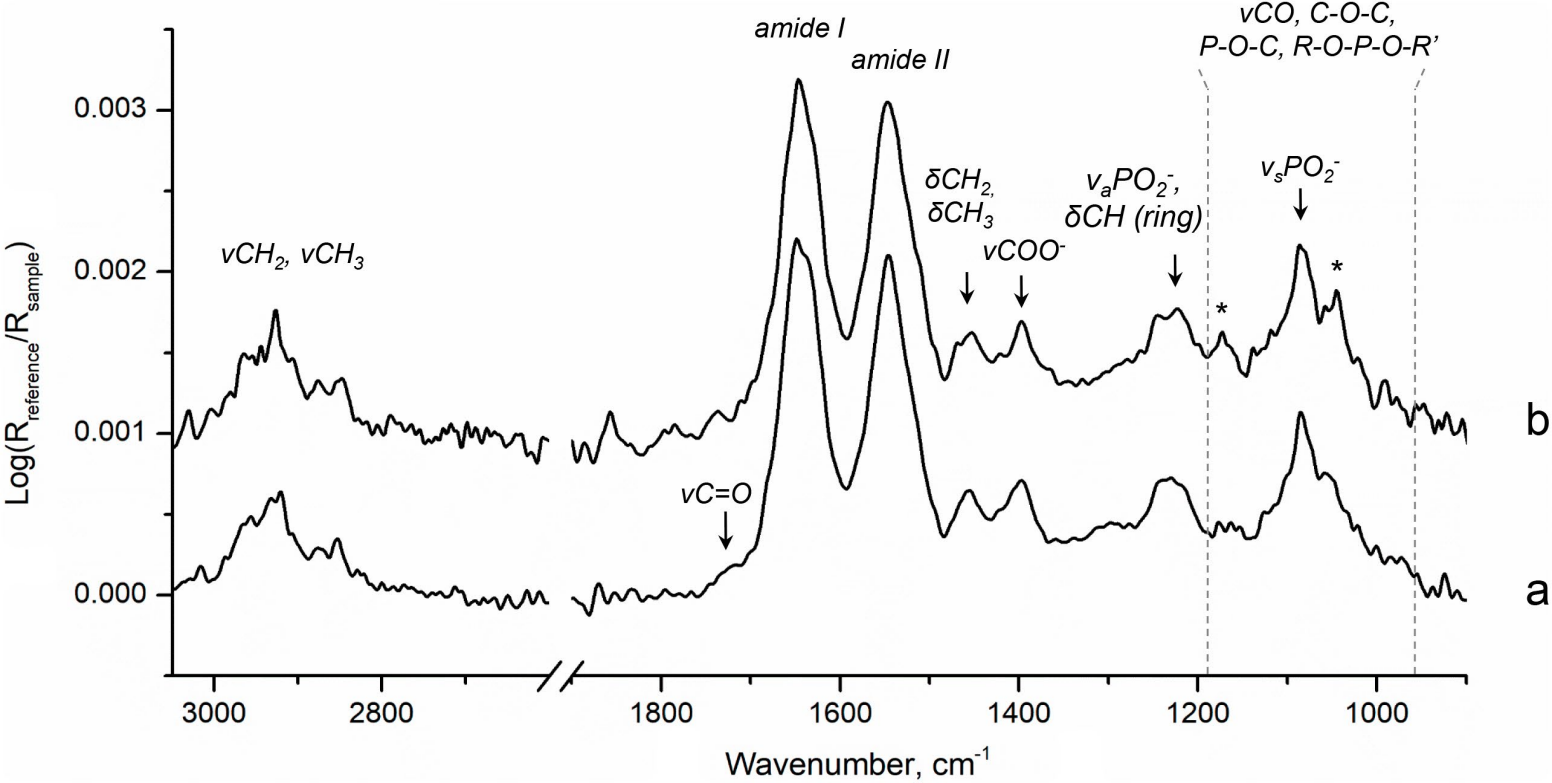
ATR-FTIR spectra of biofilms in (a) the beginning, 20 min, and (b) the end, 6 h, of Hg methylation assay. Stars indicate slight contribution of bands corresponding to MOPS buffer (Supplementary Figure S2) in spectra of biofilms.

Finally, since changes in the physiological state of the microorganism may affect the rate of MeHg formation (Goñi-Urriza et al., 2015), we investigated biochemical composition of biofilms in the beginning and at the end of the 6 h methylation assay using ATR-FTIR spectroscopy. This method is particularly sensitive to relative changes in the amounts of major biochemical compounds such as proteins, polysaccharides, nucleic acids and lipids, which can be monitored at native state. Nonetheless, some specific metabolic changes can also be detected upon changes in the cell environment. For example, in *G. sulfurreducens* planktonic cultures we observed significant variation in relative amounts of energy-reserve compounds and nucleic acids during Hg methylation assays that were consistent with changes in cell density (Gutensohn et al., unpublished data). The ATR-FTIR spectra of biofilms showed that relative amounts of proteins (indicated by amide I and amide II bands), nucleic acids (ν_a_PO_2_^−^, ν_s_PO_2_^−^, δCH), lipids (CH_2_, CH_3_) and polysaccharides (νCO, C–O–C, P–O–C, R–O–P–O–R′) were similar at both time points (Bremer and Geesey, 1991; Stuart, 2004; Yunda and Quilès, 2019; Figure 5). Furthermore, the stable relative content of nucleic acids, indicated mainly by the bands at 1240–1220 cm^−1^, suggests that the cell number in biofilms likely remained unchanged throughout the assay. Hence, in accordance with the design of the assay, the experimental conditions allowed limiting cells growth and strong changes in cells physiology.

## 4. Conclusion

In this work, we demonstrate that *G. sulfurreducens* biofilms prepared in media with various nutrient load produce MeHg, from which a significant portion is released in the surrounding medium. The addition of vitamins during the growth allowed for significantly higher biofilm surface coverage 3 days of cultivation, compared to other media investigated. The biofilm formed was characterized by generally high rate of MeHg formation, although this value was three to five times lower than the rates of MeHg formation in assays with *G. sulfurreducens* planktonic cultures found in our and previous works. The percent of Hg(II) associated with cells in the biofilm was notably lower than the values found for planktonic cultures, while the fraction of MeHg of total Hg in the biofilm was remarkably high. The high fraction of MeHg in biofilms and low Hg(II) accumulation in biofilms suggest that *G. sulfurreducens* rapidly transforms Hg(II) into MeHg once Hg(II) reaches the biofilm.

## Data availability statement

The original contributions presented in the study are included in the article/Supplementary material, further inquiries can be directed to the corresponding author/s.

## Author contributions

The study was designed and planned by EY, MR, and EB. The experimental work was performed by EY, with the contribution from MG. Data analysis was performed and the first draft of the manuscript was compiled by EY. All authors contributed to the article and approved the submitted version.

## Funding

The Kempe Foundation is acknowledged for funding (JCK-1917).

## Conflict of interest

The authors declare that the research was conducted in the absence of any commercial or financial relationships that could be construed as a potential conflict of interest.

## Publisher’s note

All claims expressed in this article are solely those of the authors and do not necessarily represent those of their affiliated organizations, or those of the publisher, the editors and the reviewers. Any product that may be evaluated in this article, or claim that may be made by its manufacturer, is not guaranteed or endorsed by the publisher.
