# Supplementary material for: Methylmercury formation in biofilms of Geobacter sulfurreducens
Source: Front Microbiol. 2023 Jan 13;14:1079000. doi: 10.3389/fmicb.2023.1079000 (PMC9880215; doi:10.3389/fmicb.2023.1079000)
Supplement: Supplementary file 1 [file Data_Sheet_1.PDF]

Supporting information

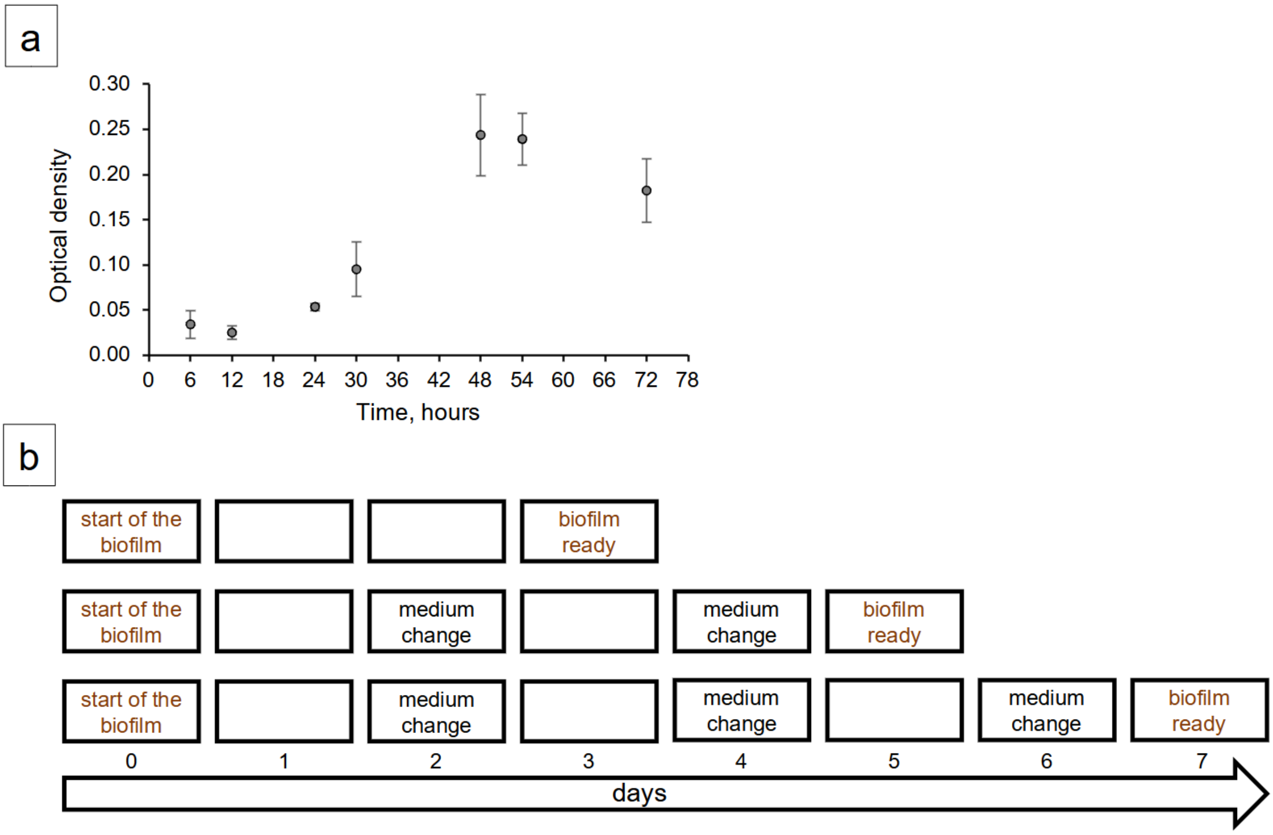

Figure SI1. Preparation of G. sulfurreducens biofilms: a) time evolution of the optical density of G. sulfurreducens planktonic culture in the standard nutritive medium, b) schematic representation of the procedure for obtaining 3-, 5- or 7-day-old biofilms

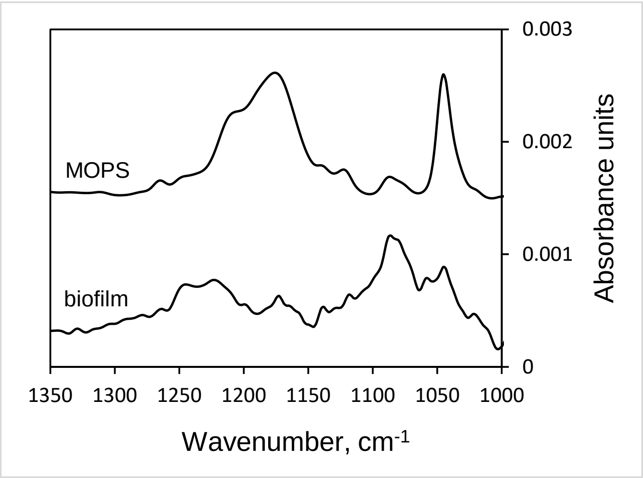

Figure SI2. ATR-FTIR spectra of MOPS aqueous solution and G. sulfurreducens biofilm pellet suspended in the assay medium containing MOPS. A slight contribution of MOPS in the biofilm spectrum can be seen at around 1050 and 1175 cm^{-1} due to progressive adsorption of MOPS on the ATR-crystal during spectrum recording.
